# Clinical characteristics and outcomes of anti-NMDA receptor-dominant autoimmune encephalitis

**DOI:** 10.3389/fneur.2026.1844969

**Published:** 2026-05-26

**Authors:** Ming Guo, Dongmei Xu

**Affiliations:** 1 Department of Neurology, Xi'an Eighth Hospital, Xi'an, China; 2 Department of Neurology, Yan'an University Xianyang Hospital, Xianyang, China

**Keywords:** encephalitis, anti-NMDA receptor encephalitis, electroencephalography, magnetic resonance imaging, neuroinflammation, mechanical ventilation

## Abstract

**Introduction:**

Autoimmune encephalitis (AE) is a multifaceted neuroinflammatory condition characterized by a diverse array of neuropsychiatric symptoms and varying degrees of functional recovery.

**Methods:**

A retrospective cohort study conducted at a tertiary care institution analyzed 150 individuals diagnosed with AE from January 2017 to December 2023. The diagnosis of AE was made based on the subacute onset of neuropsychiatric symptoms that were compatible with neuronal autoantibody detection and/or distinctive EEG or MRI findings. Analysis was done on clinical, radiological, electrophysiological, and serological data. Clinical outcomes were assessed using the modified Rankin Scale (mRS) at the final follow-up appointment, with a score of ≥3 indicating poor outcome. Multivariate logistic regression was employed to identify independent predictors of a poor prognosis.

**Results:**

The average age of the cohort was 34.0 ± 7.7 years, with 63 (42.0%) testing positive for anti-NMDA receptor antibodies. The most prevalent clinical manifestations included mental disorders 103 (68.7%), cognitive impairment 102 (68.0%), and seizures 89 (59.3%). EEG abnormalities 95 (63.3%) were more frequent than MRI abnormalities 57 (38.0%). Poor outcomes were observed in 45 (30.0%) of patients. Multivariate analysis identified four independent predictors of poor outcomes: mechanical ventilation (OR 10.72, 95% CI: 2.97-38.70, *p* < 0.001), presence of a tumor (OR 8.42, 95% CI: 2.07-34.24, *p* = 0.003), impaired consciousness (GCS < 8) (OR 4.05, 95% CI: 1.26-13.07, *p* = 0.019), and increasing age (OR 1.43, 95% CI: 1.26-1.63, *p* < 0.001). These results should be interpreted within this antibody distribution due to the prevalence of anti-NMDAR patients and the lack of thorough antibody stratification.

**Discussion:**

In conclusion, advanced age, underlying malignancy, mechanical ventilation, and severe consciousness impairment are significant independent factors associated with poor functional recovery in autoimmune encephalitis.

## 1 Introduction

Autoimmune encephalitis (AE) is a neuroinflammatory condition that presents a wide range of clinical symptoms, from mild cognitive issues to severe pan-encephalitis (1, 2). Initially associated with critically ill patients showing subacute cognitive decline, seizures, or status epilepticus, it is now understood that AE can impact various regions of the brain beyond just the limbic system (3, 4).

Autoimmune encephalitis (AE) is characterized by an immune response in which autoantibodies mistakenly target neural components as foreign antigens (5). This immune attack is triggered by antibodies that target cell-surface receptors, ionic channels, synaptic proteins, and intracellular onconeural antigens. Anti-NMDA receptor antibodies are the most prevalent subtype, but other well-known antibodies include LGI1, CASPR2, AMPA receptor, GABAB receptor, and intracellular antibodies like Hu and Ma2. Significantly, certain of these antibody subtypes are linked to small-cell lung carcinoma, thymoma, breast or testicular tumors, and underlying cancers, most frequently ovarian teratomas. This leads to neuroinflammation and potential damage to the brain’s functional structure (1, 5).

In modern frameworks, autoimmune encephalitis is characterized by a polysymptomatic nature that spans a broad spectrum of neurological and psychiatric symptomatology. Different autoantibodies can cause distinct core symptoms based on the affected protein, but the overall presentation is diverse, including psychiatric symptoms and various movement disorders. This complexity makes it difficult to classify clinically and highlights the need for identifying key predictors to determine appropriate treatment strategies (6). It is critical to understand that autoimmune encephalitis is a complex disease. Treatment regimens should be tailored to each patient’s symptoms, antibody results, and response to therapy. For many people, early identification and treatment can result in significant symptom relief and a better prognosis (Graus et al., 2016). However, some cases may be more difficult to cure, resulting in long-term neurological consequences. As a result, persons suffering from this illness require specialized treatment and continuing medical monitoring (5).

Receiving treatment in the intensive care unit (ICU) is a significant risk factor for a negative long-term prognosis in patients with acute encephalitis (AE). Approximately 55% of AE cases require ICU care (7), which is associated with increased mortality rates ranging from 18.5 to 40% (8). Other factors that contribute to a poor outcome include the severity of the disease, lack of response to standard immunotherapies, length of ICU stay, frequency of complications in the ICU, delay in initiating treatment, presence of comorbidities, and age (9, 10). The prognosis of paraneoplastic syndromes varies widely depending on the subtype. Patients with cell surface antibodies tend to have better outcomes, while those with classic paraneoplastic cases targeting intracellular antigens often have poor outcomes. However, even within subtypes, there are significant inconsistencies in long-term morbidity (11). It is important to understand the factors that may influence prognosis to provide valuable information to clinicians, patients, and families, and to potentially impact future treatment decisions.

Despite the increasing clinical understanding of autoimmune encephalitis, there is still a significant knowledge gap regarding the predictive power of baseline clinical variables. While factors such as age and malignancy are commonly mentioned in case reports, there is a lack of substantial, multivariable research that establishes their independent risk (odds ratios) for long-term disability. This study aims to address this gap by conducting a thorough statistical analysis of 150 patients to determine which baseline markers; specifically age, GCS scores, and underlying malignancy—most accurately predict poor functional outcomes. Identifying these associations is essential for moving away from a one-size-fits-all approach and toward a more precise risk-stratification model in neurocritical care.

## 2 Methodology

### 2.1 Study design

This retrospective cohort investigation was carried out at a tertiary care hospital to assess the clinical features and outcomes of individuals diagnosed with autoimmune encephalitis, including all consecutive patients hospitalized over a specific study period from January 2017 to December 2023.

### 2.2 Study population

There were 150 patients with an autoimmune encephalitis diagnosis. Graus et al. (2016) defined diagnostic criteria that were used to identify cases. Patients were specifically included if they had at least one of the following: new-onset seizures, movement disorders, or diminished consciousness, in addition to subacute onset (≤3 months) of important symptoms including psychiatric problems. Seronegative and antibody-positive patients were both included. Anti-NMDA receptor antibodies were used to identify antibody-positive AE. When seronegative patients satisfied clinical criteria and were confirmed by abnormal EEG and/or MRI results, they were categorized as probable AE. Only individuals with a verified clinical diagnosis of autoimmune encephalitis and comprehensive records of important factors (antibody status, neuroimaging, EEG, and clinical presentation) were included. Furthermore, the inclusion of patients was limited to those having documented follow-up for outcome evaluation (mRS). Cases with inadequate records or missing outcome data were also removed, as were patients with alternative diagnoses such as infectious encephalitis, metabolic or toxic encephalopathy, or main psychiatric problems.

### 2.3 Data collection

A structured data collecting form was used to retrieve data from medical records. Age and sex were among the baseline demographic factors. Prodromal symptoms, psychiatric symptoms, seizures, cognitive impairment, mobility problems, and disturbance of consciousness were among the clinical features noted upon presentation. The Glasgow Coma Scale (GCS) was used to measure consciousness, and a score of less than eight was considered serious impairment. Deficits in higher cortical functions (such as memory, attention, and orientation) in conscious patients as reported in clinical records based on bedside neurological examination were characterized as cognitive impairment. According to clinical evaluations by neurologists and/or psychiatrists, psychiatric abnormalities were characterized as the presence of symptoms including agitation, hallucinations, delusions, mood disorders, or behavioral changes. The Glasgow Coma Scale (GCS) was used to measure disturbance of consciousness; a score of less than eight indicated serious impairment (coma). Additionally, indicators of the severity of the illness were recorded, such as the need for mechanical ventilation. The existence of an underlying tumor, magnetic resonance imaging (MRI) findings, EEG outcomes, and anti-NMDA receptor antibody serological status were other variables. Neuronal autoantibody serological testing was done as part of the standard clinical assessment. According to normal diagnostic procedure, anti-N-methyl-D-aspartate receptor (NMDAR) antibodies were detected in blood and/or cerebrospinal fluid (CSF) using cell-based assays (CBA).

### 2.4 Treatment characteristics

Medical records were also used to extract information on treatment modalities, such as first-line immunotherapy (high-dose corticosteroids, intravenous immunoglobulin, and/or plasma exchange), second-line therapies (cyclophosphamide or rituximab), tumor-directed interventions when appropriate, and supportive care like mechanical ventilation.

### 2.5 Outcome assessment

The modified Rankin Scale (mRS) was used to evaluate long-term outcome at the last documented follow-up. The follow-up period lasted 6 months. To ensure consistency and reliability, two independent neurologists with formal training in standardized mRS evaluation and prior clinical experience in neurocritical care evaluated the mRS scores. In situations where there were disagreements, a consensus score was established through discussion. Due to the study’s retrospective design, a thorough analysis of medical records, including recorded neurological exams and follow-up clinic notes, was the main basis for mRS assessment. Individuals were split into two categories: those with a favorable outcome (mRS ≤ 2) and those with poor outcome (mRS ≥ 3). This classification was utilized to compare individuals with functional recovery versus those with severe disability.

### 2.6 Statistical analysis

Statistical software IBM SPSS Statistics (version 26.0) was used for the quantitative analysis. The mean ± standard deviation was used to represent continuous variables, whereas frequencies and percentages were employed to depict categorical variables. The Shapiro–Wilk test was used to evaluate the normality of continuous data (age). Suitable statistical tests were used to do evaluations between outcome groups.

For continuous factors, independent sample *t*-tests were used, and chi-square tests were applied for categorical factors. To find independent predictors of a negative outcome, variables showing possible connections (*p* < 0.05) in univariate analysis were added to a multivariate logistic regression analysis. Adjusted odds ratios (ORs) with 95% confidence intervals (CIs) were computed. Statistical significance was defined as a *p*-value of <0.05.

### 2.7 Handling of missing data

As specified in the inclusion criteria, only patients with complete records for important research variables were included in the final analysis when it came to missing data. As a result, analyses were carried out on a complete-case basis and no imputation techniques were used.

## 3 Results

### 3.1 Baseline clinical characteristics

**Figure 1:**
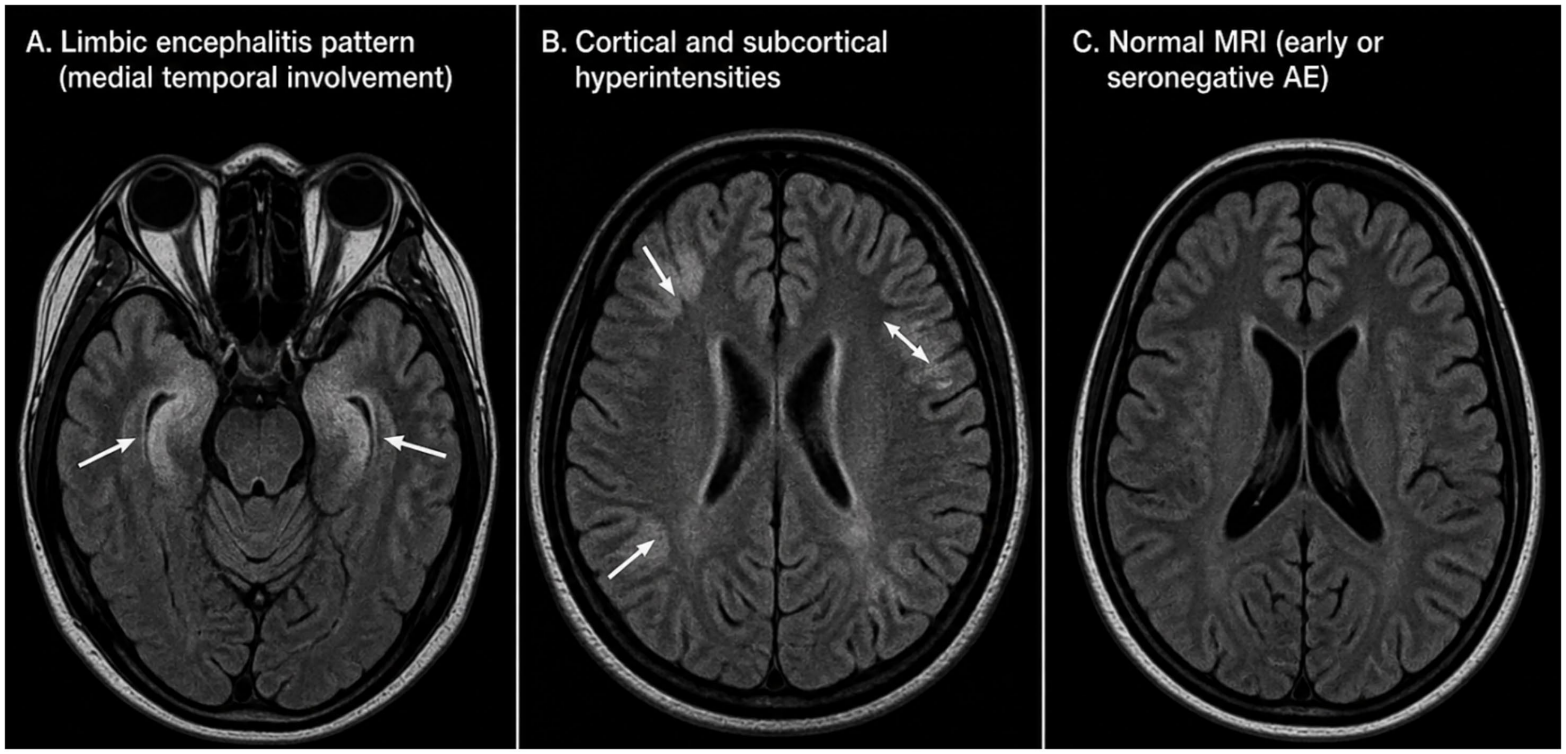
Representative MRI findings in autoimmune encephalitis. **(A)** Limbic encephalitis pattern demonstrating bilateral mesial temporal (hippocampal) hyperintensities on axial FLAIR imaging (arrows). **(B)** Non-limbic involvement with patchy cortical and subcortical hyperintensities in extra-temporal regions (arrows), suggesting diffuse neuroinflammatory alterations. **(C)** Normal-appearing MRI without focal signal abnormality. Normal imaging does not rule autoimmune encephalitis and is frequently seen in early-stage illness or anti-NMDAR encephalitis.

The investigation comprised 150 autoimmune encephalitis cases in total. The average age was 34.0 ± 7.7 years, and 71 (47.3) of individuals were male. Sixty-seven percent of patients had prodromal symptoms. Cognitive impairment and psychiatric symptoms were noted in 68.0 and 68.7% of individuals, respectively. Fifty-nine percent of patients had seizures. Disturbance of consciousness (GCS < 8) was observed in 53 (35.3) of individuals. 29 (19.3) of subjects had movement disorders. 47 (31.3) of patients needed mechanical ventilation. 29 (19.3) of patients were found to have tumors (most commonly ovarian teratoma). EEG abnormalities were found in 95 (63.3) of patients, while MRI abnormalities were recorded in 57 (38.0) of cases. Further characterization revealed that T2/FLAIR hyperintensities affecting the medial temporal lobes (limbic areas) constituted the majority of MRI abnormalities, with additional cortical and subcortical signal alterations noted in a subgroup of patients (Figure 1).

Diffuse or localized slowness, epileptiform discharges, and uncommon severe delta brush patterns were the primary characteristics of EEG abnormalities. Based on recognized clinical criteria validated by EEG and/or MRI results, 63 patients (42.0%) had seropositive anti-NMDA receptor antibodies, while the remaining 87 patients (58.0%) had seronegative autoimmune encephalitis. At follow-up, long-term outcomes showed that 30.0% of patients experienced poor outcomes (mRS ≥ 3) (Table 1).

**Table 1:** Baseline clinical characteristics.

Variable	Value
Age (years)	34.0 ± 7.7
Male sex, *n* (%)	71 (47.3)
Prodromal symptoms, *n* (%)	91 (60.7)
Psychiatric symptoms, *n* (%)	103 (68.7)
Seizures, *n* (%)	89 (59.3)
Cognitive impairment, *n* (%)	102 (68.0)
Disturbance of consciousness (GCS <8), *n* (%)	53 (35.3)
Movement disorders, *n* (%)	29 (19.3)
Mechanical ventilation, *n* (%)	47 (31.3)
Tumor present, *n* (%)	29 (19.3)
MRI abnormal, *n* (%)	57 (38.0)
EEG abnormal, *n* (%)	95 (63.3)
Anti-NMDA positive, *n* (%)	63 (42.0)
Poor outcome (mRS ≥3), *n* (%)	45 (30.0)

### 3.2 Comparison between good and poor outcome groups

According to the univariate analysis, the mean age of the individuals in the poor outcome group was higher than those in the good outcome group (41.4 ± 6.6 vs. 30.9 ± 5.7 years, *p* < 0.001). Individuals with poor outcomes were also more likely to have characteristics indicative of a more severe illness. This group had a higher prevalence of disturbance of consciousness (55.5% vs. 26.7%, *p* < 0.001). In a similar vein, individuals with poor outcomes required more mechanical ventilation (55.5% vs. 21.0%, *p* < 0.001).

Additionally, patients with poor outcomes were more likely to have tumors than those with favorable outcomes (33.3% vs. 13.3%, *p* = 0.004). Other factors such as gender, prodromal signs, psychiatric signs, seizures, cognitive impairment, movement disorders, MRI abnormalities, EEG abnormalities, and anti-NMDA receptor positivity, did not exhibit statistically significant differences between groups in univariate analysis (all *p* > 0.05) (Table 2).

**Table 2:** Univariate analysis.

Variable	Good outcome (*n* = 105), (mean ± SD, *n* (%))	Poor outcome (*n* = 45), (mean ± SD, *n* (%))	*p*-value
Age (years)	30.9 ± 5.7	41.4 ± 6.6	<0.001
Male sex	51 (48.6)	20 (44.4)	0.643
Prodromal symptoms	67 (63.8)	24 (53.3)	0.229
Psychiatric symptoms	70 (66.7)	33 (73.3)	0.420
Seizures	60 (57.1)	29 (64.4)	0.404
Cognitive impairment	73 (69.5)	29 (64.4)	0.541
Disturbance of consciousness (GCS <8) (%)	28 (26.7)	25 (55.5)	<0.001
Movement disorders	21 (20.0)	8 (17.8)	0.752
Mechanical ventilation	22 (21.0)	25 (55.5)	<0.001
Tumor present	14 (13.3)	15 (33.3)	0.004
MRI abnormal	38 (36.2)	19 (42.2)	0.486
EEG abnormal	68 (64.8)	27 (60.0)	0.579
Anti-NMDA	45 (42.9)	18 (40.0)	0.745

### 3.3 Multivariate analysis

A multivariate logistic regression analysis was used to find variables that were independently associated to a poor outcome. Age continued to be a significant predictor of poor outcome (adjusted OR 1.43, 95% CI 1.26–1.63, *p* < 0.001). Presence of tumor was independently related with poor outcome (adjusted OR 8.42, 95% CI 2.07–34.24, *p* = 0.003). Additionally, mechanical ventilation was significantly associated with poor outcomes (adjusted OR 10.72, 95% CI 2.97–38.70, p < 0.001). Lastly, consciousness disturbance continued to be independently connected to a poor outcome (adjusted OR 4.05, 95% CI 1.26–13.07, *p* = 0.019) (Table 3).

**Table 3:** Multivariate logistic regression.

Variable	Adjusted OR	95% CI	*p*-value
Age	1.43	1.26–1.63	<0.001
Tumor presence	8.42	2.07–34.24	0.003
Mechanical ventilation	10.72	2.97–38.70	<0.001
Disturbance of consciousness (GCS < 8)	4.05	1.26–13.07	0.019

Overall, these results show that although autoimmune encephalitis presents with a variety of clinical characteristics, patient-associated variables like growing older and the presence of a tumor appear to be particularly associated with adverse outcomes, as do indicators of greater disease severity, especially impaired consciousness and the need for mechanical ventilation.

## 4 Discussion

In this study, we conducted a retrospective analysis of the clinical records of 150 patients diagnosed with autoimmune encephalitis to determine the early predictors of long-term recovery. Our results indicate that a significant proportion of patients achieve functional independence, but around 30.0% experience a poor outcome, with persistent disability at their last follow-up.

The majority of the patients in our sample had anti-NMDA receptor encephalitis, which makes it difficult to project results to the entire range of autoimmune encephalitis. Because cell-surface and intracellular antibody-mediated syndromes are known to have different prognoses, the lack of thorough antibody stratification may mask subtype-specific effects. Additional clinically significant antibodies include intracellular onconeural antibodies like Hu and Ma2, as well as LGI1, CASPR2, AMPAR, and GABAB receptor antibodies. Consequently, this antibody distribution should be taken into consideration when interpreting the results.

To stratify outcomes, reliable prognostic instruments have been presented, such as the anti-NMDAR Encephalitis One-Year Functional Status (NEOS) score, which takes into account variables including ICU hospitalization, treatment delay, and lack of early clinical improvement. Although our approach finds overlapping disease severity signals, using such standardized tools could improve prognosis accuracy even more.

Our research indicates that the use of mechanical ventilation is the strongest factor influencing long-term disability. The need for life support serves as a key indicator of a more severe neuro-immunological profile, with disruptions in autonomic breathing functions suggesting significant deep-brain damage that leads to a poor functional outcome.

This respiratory failure is often linked to a specific mechanism called central hypoventilation. Study by Xu et al. (12) focusing on anti-NMDAR encephalitis, have shown that the antibodies in this condition target the “breathing centers” in the medulla, the lower part of the brain. When these centers are affected, specifically the NMDA receptors that regulate the automatic rhythm of respiration—the brain fails to maintain a respiratory rhythm, especially during sleep.

Our results also align with those of Qin et al. (13), who conducted a retrospective cohort study involving 134 adult patients specifically admitted to the neurological ICU for severe forms of autoimmune encephalitis. Their investigation particularly concentrated on critically ill subjects and used long-term functional outcomes at 12 months, showing that mechanical ventilation was an independent predictor of poor prognosis (*p* = 0.025). In their high-severity group, 67.6% of patients with poor outcomes needed ventilatory support, a proportion similar to the 55.5% observed in our subgroup with poor outcomes. This consistency may indicate the need for a ventilator, serves as a reliable clinical indicator of an aggressive disease phenotype that often results in long-term disability.

Our research shows that the presence of an underlying tumor is a significant independent predictor of poor recovery (OR 8.42). This suggests that an oncological factor leads to a shift toward persistent, T-cell mediated neuronal damage rather than the reversible synaptic dysfunction observed in non-paraneoplastic cases. Bost et al. (14) further elaborate on this prognostic challenge in their study of 252 patients from the French Paraneoplastic Neurological Syndrome Reference Center. They found that while the neurological symptoms were similar across different types of tumors, the presence of a malignant tumor was associated with a significantly higher risk of mortality (86% versus 2% in non-malignant cases, *p* < 0.001).

The connection between malignancy and impaired functional recovery is mediated by a strong peripheral immune response. Tumor-associated antigens stimulate T cells, leading to a systemic immune attack that can initially control cancer growth but also triggers an immune response against neural antigens in the central nervous system (15). In our study, the 8.42-fold higher risk of poor outcomes may be due to this dual-target immune response, where the tumor serves as a continuous source of antigens that sustain neuroinflammation even when the cancer is not clinically evident or is small in size.

Our research demonstrates that older age is a key independent predictor, suggesting that reduced neural reserve and age-related neurodegenerative processes may contribute to a more challenging disease progression compared to the reversible synaptic mechanisms observed in younger individuals. This increased risk of poor outcomes per year of age aligns with a study at the Mayo Clinic by Kunchok et al. (16). In this broad retrospective laboratory-based investigation of 42,032 subjects, the most prevalent AE antibodies were NMDA-R-IgG, GAD65-IgG, LGI1-IgG, and MOG-IgG1, with age and sex trends indicated developmental and aging-associated (like paraneoplastic) effects on autoimmune encephalitis.

Moreover, Age, gender, CSF/MRI abnormalities, and status epilepticus showed little predictive significance, but delayed or no immunotherapy, altered consciousness, and ICU admission were determined to indicate poor outcomes in a systematic review of autoimmune encephalitis (44 studies; 2,823 individuals). Nevertheless, the overall evidence was constrained by significant heterogeneity in study design, sample size, and study quality (17).

Our study shows that impaired consciousness upon admission is a strong independent predictor, suggesting that the severity of coma at the beginning is associated with a higher level of neuro-inflammatory activity and more widespread cortical dysfunction compared to patients with only psychiatric or cognitive symptoms (OR 4.05). This result aligns again with systematic review conducted by Broadley et al. (17), which highlighted altered consciousness upon admission as a reliable indicator of long-term disability. The prognostic value of this factor is supported by the pathophysiological insights provided by Wandinger et al. (18), who describe autoimmune encephalitis as a progressive disorder with distinct stages. Their research suggests that the transition from initial psychiatric symptoms to impaired wakefulness signifies a critical worsening of the condition, marked by synaptic signal transduction impairment and escalating neuroinflammation.

We found that individuals with impaired consciousness are at a 4.05-fold increased risk, indicating that they may have advanced beyond the early reversible stages of the disease. This often requires prolonged intensive care. Our findings align with Wandinger et al.’s stage-specific framework, highlighting that impaired consciousness is a clear indicator of advanced neuroimmunological involvement. Therefore, the presence of impaired consciousness should prompt immediate escalation of immunotherapy to reduce the risk of permanent cognitive and functional impairments.

### 4.1 Strengths

- This study, with a sample size of 150 patients, offers substantial statistical power for a rare neuroimmunological condition, enabling strong multivariable analysis.
- Key risk factors such as Mechanical and Tumor Presence were identified, serving as important clinical indicators for predicting poor prognosis.
- The results regarding impaired wakefulness are consistent with the multistage model proposed by Wandinger et al. (2018), supporting our data with established pathophysiological stages.

### 4.2 Limitations

- Due to the retrospective nature of our study, we rely on existing medical records and cannot establish a clear causal timeline for all symptoms.
- The results should be interpreted cautiously when applied to other situations or groups because this is a single-center retrospective study with limited generalizability.
- This study was conducted at a tertiary center, which may introduce a bias toward more severe or difficult cases due to referral patterns.
- The rarity of certain intracellular antibody subtypes limited our ability to conduct a detailed analysis based on specific antigens.
- Patients with incomplete records may have been excluded due to the demand for complete clinical and follow-up data, which may have induced selection bias.
- This study did not assess differences in tumor management and immunotherapy strategies, which might have affected patient outcomes.

## 5 Conclusion

This study highlights that early illness severity and related systemic variables are the main determinants of outcomes in autoimmune encephalitis. Patients at the highest risk of a poor functional recovery are consistently identified by advanced age, underlying cancer, the necessity for mechanical ventilation, and substantial impairment of consciousness. In order to provide prompt, aggressive immunotherapy, careful monitoring, and multidisciplinary care, early identification of these high-risk characteristics is crucial. By incorporating these variables into standard clinical evaluation, prognosis accuracy can be improved and more focused management approaches that aim to improve neurological outcomes can be supported. To improve predictive frameworks and create focused therapies meant to lower morbidity and improve functional recovery in this complicated condition, more extensive prospective studies are necessary.

## Data Availability

The raw data supporting the conclusions of this article will be made available by the authors, without undue reservation.

## References

[1] Graus F Titulaer MJ Balu R Benseler S Bien CG Cellucci T . A clinical approach to diagnosis of autoimmune encephalitis. Lancet Neurol. (2016) 15:391–404. doi: 10.1016/S1474-4422(15)00401-9 26906964 PMC5066574

[2] Bastiaansen AE De Bruijn MA Schuller SL Martinez-Hernandez E Brenner J Paunovic M . Anti-NMDAR encephalitis in the Netherlands, focusing on late-onset patients and antibody test accuracy. Neurol Neuroimmunol Neuroinflamm. (2021) 9:1127. doi: 10.1212/nxi.0000000000001127 PMC8696553 34937737

[3] Dalmau J Armangué T Planagumà J Radosevic M Mannara F Leypoldt F . An update on anti-NMDA receptor encephalitis for neurologists and psychiatrists: mechanisms and models. Lancet Neurol. (2019) 18:1045–57. doi: 10.1016/s1474-4422(19)30244-3 31326280

[4] Bastiaansen AE Van Steenhoven RW De Bruijn MA Crijnen YS Van Sonderen A Van Coevorden-Hameete MH . Autoimmune encephalitis resembling dementia syndromes. Neurol Neuroimmunol Neuroinflamm. (2021) 8:1039. doi: 10.1212/nxi.0000000000001039 PMC8362342 34341093

[5] Patel A Meng Y Najjar A Lado F Najjar S. Autoimmune encephalitis: a physician’s guide to the clinical spectrum diagnosis and management. Brain Sci. (2022) 12:1130. doi: 10.3390/brainsci12091130, 36138865 PMC9497072

[6] Uy CE Binks S Irani SR. Autoimmune encephalitis: clinical spectrum and management. Pract Neurol. (2021) 21:412–23. doi: 10.1136/practneurol-2020-002567, 34108243 PMC8461404

[7] Yeshokumar AK Gordon-Lipkin E Arenivas A Cohen J Venkatesan A Saylor D . Neurobehavioral outcomes in autoimmune encephalitis. J Neuroimmunol. (2017) 312:8–14. doi: 10.1016/j.jneuroim.2017.08.010, 28889962

[8] Harutyunyan G Hauer L Dünser MW Karamyan A Moser T Pikija S . Autoimmune encephalitis at the neurological intensive care unit: etiologies, reasons for admission and survival. Neurocrit Care. (2016) 27:82–9. doi: 10.1007/s12028-016-0370-7, 28028790 PMC5524849

[9] Chi X Wang W Huang C Wu M Zhang L Li J . Risk factors for mortality in patients with anti-NMDA receptor encephalitis. Acta Neurol Scand. (2016) 136:298–304. doi: 10.1111/ane.12723, 28028820

[10] Schubert J Brämer D Huttner HB Gerner ST Fuhrer H Melzer N . Management and prognostic markers in patients with autoimmune encephalitis requiring ICU treatment. Neurol Neuroimmunol Neuroinflamm. (2018) 6:e514. doi: 10.1212/nxi.0000000000000514, 30568992 PMC6278855

[11] Aurangzeb S Symmonds M Knight RK Kennett R Wehner T Irani SR. LGI1-antibody encephalitis is characterised by frequent, multifocal clinical and subclinical seizures. Seizure. (2017) 50:14–7. doi: 10.1016/j.seizure.2017.05.017, 28586706 PMC5558811

[12] Xu Q Wang Q Han J Mao F Zeng S Chen S . Central hypoventilation is a key risk factor for mechanical ventilation during the acute phase of anti-N-methyl-D-aspartate receptor encephalitis. Front Neurol. (2021) 12:728594. doi: 10.3389/fneur.2021.728594, 34795627 PMC8594565

[13] Qin L Chen K Zhou Y Wang W Lu W Zhang H. Clinical features and outcomes in adult patients with autoimmune encephalitis requiring intensive care: a retrospective cohort study. Neurocrit Care. (2025) 44:273–81. doi: 10.1007/s12028-025-02374-2, 40958052 PMC12819487

[14] Bost C Chanson E Picard G Meyronet D Mayeur M Ducray F . Malignant tumors in autoimmune encephalitis with anti-NMDA receptor antibodies. J Neurol. (2018) 265:2190–200. doi: 10.1007/s00415-018-8970-0, 30003358

[15] Dalmau J Graus F. Antibody-mediated encephalitis. N Engl J Med. (2018) 378:840–51. doi: 10.1056/nejmra1708712 29490181

[16] Kunchok A McKeon A Zekeridou A Flanagan EP Dubey D Lennon VA . Autoimmune/paraneoplastic encephalitis antibody biomarkers: frequency, age, and sex associations. Mayo Clin Proc. (2021) 97:547–59. doi: 10.1016/j.mayocp.2021.07.023, 34955239

[17] Broadley J Seneviratne U Beech P Buzzard K Butzkueven H O’Brien T . Prognosticating autoimmune encephalitis: a systematic review. J Autoimmun. (2018) 96:24–34. doi: 10.1016/j.jaut.2018.10.014 30595145

[18] Wandinger KP Leypoldt F Junker R. Autoantibody-Mediated Encephalitis. Deutsches Arzteblatt international. (2018) 115:666–673. doi: 10.3238/arztebl.2018.0666 30381132 PMC6234470

